# Manipulation of DNA Repair Proficiency in Mouse Models of Colorectal Cancer

**DOI:** 10.1155/2016/1414383

**Published:** 2016-06-20

**Authors:** Michael A. Mcilhatton, Gregory P. Boivin, Joanna Groden

**Affiliations:** ^1^Department of Cancer Biology and Genetics, The Ohio State University, 460 West 12th Avenue, Columbus, OH 43210, USA; ^2^Department of Pathology, Boonshoft School of Medicine, Wright State University, Health Sciences Building 053, 3640 Colonel Glenn Highway, Dayton, OH 45435, USA

## Abstract

Technical and biological innovations have enabled the development of more sophisticated and focused murine models that increasingly recapitulate the complex pathologies of human diseases, in particular cancer. Mouse models provide excellent* in vivo* systems for deciphering the intricacies of cancer biology within the context of precise experimental settings. They present biologically relevant, adaptable platforms that are amenable to continual improvement and refinement. We discuss how recent advances in our understanding of tumorigenesis and the underlying deficiencies of DNA repair mechanisms that drive it have been informed by using genetically engineered mice to create defined, well-characterized models of human colorectal cancer. In particular, we focus on how mechanisms of DNA repair can be manipulated precisely to create* in vivo* models whereby the underlying processes of tumorigenesis are accelerated or attenuated, dependent on the composite alleles carried by the mouse model. Such models have evolved to the stage where they now reflect the initiation and progression of sporadic cancers. The review is focused on mouse models of colorectal cancer and how insights from these models have been instrumental in shaping our understanding of the processes and potential therapies for this disease.

## 1. The Mouse as a Model Organism for Colorectal Cancer Studies

The study of cancer biology advances continually and generates complex emergent data. In the area of biological sciences, technology has arguably outpaced our ability to fully interpret the wealth of available data and subsequent implications for understanding cancer pathogenesis [1]. Evolving platforms for sequence analyses, expression arrays, and proteomic and metabolomic characterization of tumor tissues relentlessly refine our resolution of the crucial biological processes inherent to the initiation and progression of human cancers [2].

The development of more effective therapeutic modalities for cancer treatment remains a driving priority of modern biomedical science. This imperative requires appropriate models to provide conceptual frameworks for deciphering the various biological pathways that collaborate in the initiation and progression of human cancers. Ideally these models will mimic the complexity of cancer development and provide a biological system for both identifying and assessing relevant therapeutic targets [3, 4]. The mouse presents a useful animal surrogate for unraveling the complexities of human tumor biology in an* in vivo* setting. Furthermore, the genomic sequences of common laboratory strains have been determined, revealing the high degree of conservation between mouse genes and their cognate human counterparts [5]. Mouse models have made tremendous contributions to our understanding of the pathologies of many diseases, including cancer [3, 4], but a comprehensive evaluation is beyond the scope of this review. Instead, we will confine this discussion to the utility of the mouse as a model for studying colorectal cancer, a focus of our laboratory for many years.

There are significant gaps in our ability to predict the inherited risk of developing colorectal cancer and in our understanding of the biological mechanisms that lead to its initiation and progression. Colorectal cancer is the second leading cause of cancer-related death in the Western world and is currently the third most common form of cancer. Although constitutional genetics is well established as a contributor to susceptibility and screening recommendations are a well-accepted part of best clinical practices, nearly 140,000 new cases of colorectal cancer are diagnosed each year in the United States, and >50,000 attributable deaths occur annually [6]. Approximately 8 to 35% of sporadic colorectal cancer is estimated to be due to genetic variance [7–9], but genome-wide association studies (GWAS) for colorectal cancer susceptibility have only uncovered approximately 1–9% of the estimated heritable risk [9–12]. Inflammation is a known risk factor for multiple tumor types including colorectal cancer, inflammatory bowel disease (IBD), and several other conditions [13–15]. Inflammatory bowel diseases, such as Crohn's disease (CD) and ulcerative colitis (UC), are also estimated to have a high degree of heritable risk: 25–42% for CD and 4–15% for UC [16, 17]. Although more than 201 risk loci for IBD have been identified, these are estimated to only account for a little over one-third of the estimated genetic risk [18–21]. Additionally, individuals with IBD, especially those diagnosed with ulcerative colitis which specifically affects the large intestine, are at an increased risk for the development of colorectal cancer: 2% at 10 years, 8% at 20 years, and 18% at 30 years [22]. Finally, regardless of our understanding of some major risk factors for and the pathways dysregulated in colorectal cancer fifty percent of those diagnosed with localized invasive disease die within five years [6].

Mouse models are uniquely suited to test hypotheses about tumor formation in intestinal cancer* in vivo* and more than one model should be used to represent the complicated risk factors that affect tumor susceptibility within the human population. Disease pathogenesis recapitulates the adenoma-carcinoma transition of human colorectal cancer, at least at the early stages [23, 24]. Expression analyses have revealed critical similarities, and also important differences, in transcriptional profiles between various mouse models and human colorectal tumors [25].* In vivo* modeling of colorectal cancer advances a greater understanding of human tumors through insight into the cellular mechanisms that initiate and promote tumor progression. Ultimately, this knowledge can provide better patient treatment, either through more informed therapeutic interventions or through rationales which provide personalized treatments.

## 2. Genomic Instability: A Critical Element of Colorectal Cancer

The majority of colorectal cancers develop sporadically (85%), with the remaining cases arising in the context of hereditary cancer syndromes, mainly familial adenomatous polyposis coli (FAP) and Lynch syndrome, also known as hereditary nonpolyposis colon cancer (HNPCC), or against the background of inflammatory bowel disease [39]. The contribution of genomic instability to colorectal cancer has been established by numerous studies on FAP and Lynch syndrome. It was demonstrated that inactivation of the mismatch repair system (MMR) was a prerequisite for tumor development in those with Lynch syndrome [40]. The genomic instability intrinsic in tumors from these individuals is characterized by mutations at the nucleotide level, typically demonstrated by the emergence of microsatellite instability (MSI) [41, 42].

Heterogeneous deficiencies in a number of DNA repair and signaling pathways may collectively manifest as a second category of DNA instability, designated chromosomal instability (CIN), which is characterized by allelic losses, amplifications, and translocations at the chromosomal level of genomic organization. A signature phenotype of FAP is CIN, which develops as a consequence of mutations predominantly in the* APC* tumor suppressor gene [39, 43]. FAP requires the inheritance of one mutated allele of the adenomatous polyposis coli (*APC*) gene [44]. Depending on the nature of the inherited germline allele, second-hit inactivation of the wild-type allele is achieved either by loss of heterozygosity (LOH) of the (wild-type)* APC* locus or by intragenic mutation of the* APC* gene [45, 46]. APC is also inactivated by intragenic mutation in 70–80% of individuals with sporadic colorectal cancer [47, 48]. Many germline and sporadic human mutations have been mapped to codons 1250 to 1464 of the* APC* gene [47–49]. This region has been designated the mutation cluster region (MCR) and includes a mutational hotspot at codon 1309, with a second hotspot falling outside the region at codon 1061 [48, 49]. These mutations generate truncated APC proteins that lack part or all of key *β*-catenin-binding domains.

Mutation of* APC* subsequently disrupts the WNT/*β*-catenin signaling pathway [39, 43]. In the absence of* APC* mutation, alterations in *β*-catenin (*CATNB*) or other downstream genes compromise signaling in the WNT pathway [50, 51]. The variable mechanisms by which APC is targeted and the nature and distribution of the inactivating mutations themselves have led to the proposal that, dependent on mutational context, an optimal activation of WNT signaling is required for subsequent tumorigenesis [45, 46]. This is known as the “just-right” hypothesis—“just-the-right (dysregulated) level of WNT.” Persistent activation of the canonical WNT pathway in the colonic epithelium appears to be a required event to initiate subsequent adenoma formation. The pathogenic signature of FAP is revealed by the development of hundreds of small adenomatous polyps throughout the colon, a small percentage of which ultimately progress to malignant adenocarcinomas (reviewed in [39]). The inevitable outcome is colorectal cancer, mandating preemptive surgical intervention.

## 3. Models of Familial Adenomatous Polyposis Coli

**Figure 1:**
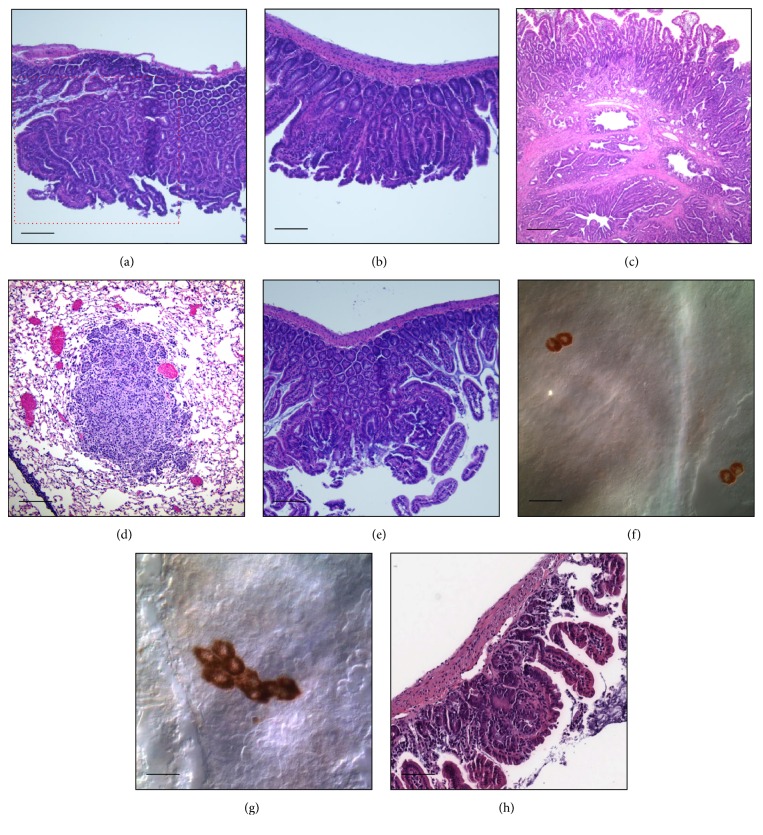
Pathology of intestinal lesions in* Apc* ^*Min/+*^ and* Blm/BLM* mice. (a) Gastrointestinal neoplasia (red box) in* Apc* ^*Min/+*^ mouse intestine; (b) adenoma in* Apc* ^*Min/+*^ mouse intestine (ileum); (c) carcinoma in* Apc* ^*Min/+*^ *;Blm* ^*Cin/+*^ mouse intestine; (d) adenoma in* Ccsp/fgf-10;Blm* ^*Cin/+*^ lung tissue; (e) adenoma in* Apc* ^*Min/+*^ *;BLM* ^*Tg*^ mouse intestine; (f) isolated retinal pigment epithelial (RPE) cells in a* p* ^*un/un*^ *;BLM* ^*Tg*^ mouse retina; (g) a cluster of RPE cells in a* p* ^*un/un*^ *;BLM* ^*Tg*^ mouse retina; and (h) adenoma in* Apc* ^*Min/*+^ *;BLM* ^*Tg*^ *;Msh2* ^Δ*7N/*Δ*7N*^ mouse intestine.

The archetypical animal model of FAP is the multiple intestinal neoplasia (Min) mouse which was originally identified following a mutagenesis screen with N-ethyl-N-nitrosourea (ENU) [26]. It was subsequently shown that the “Min” phenotype was conferred by a truncating mutation at codon 850 in the* Apc* gene. The resulting truncated Apc lacked all the motifs for interacting with *β*-catenin and consequently failed to regulate cellular levels of this protein, promoting tumorigenesis.* Apc* ^*Min*^ is embryonic lethal in the homozygous state; animals must be maintained as heterozygotes. In addition to gastrointestinal neoplasia (GIN), which is defined as “histologically apparent areas of dysplasia that are not visible grossly, <0.5–1.0 mm” [23],* Apc* ^*Min/+*^ mice develop numerous small intestinal polyps, both pedunculated and flat adenomas (Figures 1(a) and 1(b)). Tumors in this model are characterized by activated Wnt signaling [25]. The wild-type* Apc* allele is inactivated by a LOH mechanism at the locus on mouse chromosome 18 and most tumors are homozygously mutated for* Apc*. This initiating event is required for promoting tumor progression in the* Apc* ^*Min/+*^ model of intestinal neoplasia, although the wild-type allele can be inactivated by point mutations in other genetic contexts [46, 52, 53].

Min mouse tumor burden varies according to background and may even vary on the same background between different laboratories [54]. In our laboratory, we routinely observe a median of 50 polyps in the intestine of mice on a C57Bl/6J background. A number of factors, both genetic and environmental, affect intestinal polyp multiplicity in the* Apc* ^*Min/+*^ model. For example, it is well documented that* Apc* ^*Min/+*^ mice maintained on a high-fat (Western) diet develop more polyps than those on the Mediterranean diet [55, 56]. Increased dietary fat has been shown to directly increase the number and proliferation of mouse intestinal stem cells, leading to a greater incidence of spontaneous adenomas [57]. Increased dietary fat induced upregulation of PPAR-*δ* which in turn activated signaling through the Wnt/*β*-catenin pathway. Furthermore, the resident gut microflora of* Apc* ^*Min/+*^ mice influences tumorigenesis and intestinal polyp numbers; manipulating the gut microflora can effect a reduction in the overall tumor burden [58, 59]. Intestinal polyp numbers in* Apc* ^*Min/+*^ mice are also modulated by several genetic modifiers, collectively termed modifier(s) of Min (*Mom*). These may be defined genes (*Mom1*) [60] or less well-defined loci of indeterminate function such as* Mom12* and* Mom13* [61]. Compared to C57Bl/6J* Apc* ^*Min/+*^ mice, mice on a mixed C57Bl/AKR background have a reduced tumor burden of 6.0 ± 4.7 polyps [62].* Mom1*, the first reported modifier of Min, is predominantly responsible for the observed phenotype [63, 64].* Mom1* was subsequently identified as phospholipase A2 (*Pla2g2a*) [60, 65]. However, it should be noted that the role of* Mom1* is complicated by the presence of other unlinked modifiers on the AKR/J background which additionally impact polyp multiplicity [60, 63]. Other modifiers of Min are reviewed in [66].

Since the derivation of the* Apc* ^*Min/+*^ mouse, genetically engineered mice have been generated which collectively model a series of different* Apc* mutations. This series highlights the strength of the mouse as a model system, namely, the capacity to study a range of clinically related mutations under comparable* in vivo* settings. Previous models provide the rational foundations for the development of more refined models that deepen our overall understanding of how mutations in key cellular genes can give rise to tumors in humans. In all of the Apc models, loss or inactivation of the wild-type allele is required for tumor initiation and progression. Although age of onset, location, and tumor number vary according to the specific* Apc* mutation and genetic background, tumor histology is similar across the different models [23]. Relevant mutant alleles of* Apc* have been outlined in Table 1. A more exhaustive, but not necessarily comprehensive, list of* Apc* mutations, derived from the Mouse Genomic Informatics website, currently lists 26* Apc* alleles on 53 different backgrounds (http://www.informatics.jax.org/marker/phenotypes/MGI:88039). Allelic series of mutations, such as those generated for the* Apc* gene, have facilitated dissection of the key roles played by tumor suppressor and oncogenes in fundamental cellular processes and how dysregulation of such processes leads to aberrant cell growth and subsequent tumorigenesis.

A thorough discussion of the available mouse models of Apc is constrained by the limits of this review. However, it is clear that the location, as well as nature, of the inactivating mutation affects tumor incidence across different Apc models (Table 1). Variation in intestinal polyp numbers has been correlated with the location of the inactivating* Apc* mutation relative to the MCR. This has been alluded to previously in the context of the “just-right” hypothesis [45, 46]. The concomitant levels of altered Wnt signaling, for example, in* Apc* ^Δ*e1*–*15*^ [27],* Apc* ^*15lox/+*^ [28], and* Apc* ^*1322T/+*^ [29] mice, determine, at least in part, the severity and number of intestinal polyps. Although differential signaling by submaximal levels of Wnt supports the observed incidence of intestinal polyp multiplicity for several of the Apc models listed in Table 1, unfortunately it cannot account for such differences in all models. Moreover, it is worth noting that similar, or supposedly identical, models may vary in the phenotypes they present. For example,* Apc* ^*580S/+*^ [30],* Apc* ^Δ*14/+*^ [31], and* Apc* ^Δ*580/+*^ [32] mice were all engineered using similar Cre-*loxP* targeting strategies to produce truncated Apc proteins through frameshifts at codon 580. Over the course of their life spans, these animals develop approximately 7, 65, and 120 adenomas, respectively. The* Apc* ^Δ*14/+*^ and* Apc* ^Δ*580/+*^ models highlight how institutional differences, perhaps such as diet and intestinal microbiome, between independently maintained colonies can skew tumor phenotypes. The inactivating mutation in* Apc* ^*1638N/+*^ mice results in a frameshift at codon 1638 [33] whereas* Apc* ^*1638T/+*^ mice have been generated with a stop at codon 1638 [34].* Apc* ^*1638N/+*^ mice develop ~10 colonic polyploid hyperplastic lesions whereas* Apc* ^*1638T*^ develop 0 polyps. Moreover,* Apc* ^*1638T/1638T*^ mice are viable, albeit with developmental and growth abnormalities, whereas* Apc* ^*1638N/1638N*^ are embryonic lethal. The same selectable neomycin marker was used to generate both* Apc* ^*1638N/+*^ and* Apc* ^*1638T/+*^ mice, but in the latter case the marker was inserted in the sense orientation. A truncated 182 kD Apc protein could only be detected in* Apc* ^*1638T/+*^ mice; insertion of the marker in the antisense orientation abolished Apc expression in* Apc* ^*1638N/+*^ mice [34]. This is an exemplary illustration of how targeting strategy can subsequently influence the resulting phenotype of genetically engineered mouse models. Such variation of phenotypes in what should be genetically similar models could be interpreted as an inherent flaw of studying human cancers in mouse systems, but such serendipity extends and enriches the versatility of these models and presents greater opportunities for understanding the tumorigenic processes. Given the complexity and number of available Apc models and the number of potentially confounding factors, including genetic modifiers/background, composition of the intestinal microbiome, animal diet, and the possible effects of environmental parameters, such as temperature [67] on tumorigenesis, it is not surprising that phenotypic analyses and interpretation remain challenging. Regardless, the tumor pathology of the Apc mouse and its years of experimental study continue to keep it as the preferred animal model for FAP.

Mutant models of Apc have also been generated in the rat and, more recently, the pig. Polyposis in the rat colon (Pirc) model has been derived from an ENU-induced stop codon at position 1137 in the rat* Apc* gene [68]. Homozygosity for the* Apc* ^*am1137*^ mutation is embryonic lethal, similar to mouse* Apc* ^*Min*^. Heterozygous animals develop both polyps in the small intestine and colon with 100% incidence. Longer-lived* Apc* ^*am1137/+*^ rats develop adenocarcinomas. A second rat model, the Kyoto Apc Delta (KAD) rat, originates from a separate ENU mutagenesis screen [69]. This model also contains a stop codon, but at Apc position 2523 that, in contrast to the Pirc rat, retains the *β*-catenin-binding region of the protein. Unlike the Pirc rat, animals homozygous for* Apc* ^Δ*2523*^ are viable and do not develop spontaneous intestinal tumors. However, they are highly sensitive to AOM/DSS induced colitis-associated colon carcinogenesis. Gene targeting, rather than mutagenesis by ENU, has generated germline stop codons at both positions in* APC* ^*1061*^ and A*PC* ^*1311*^ cloned pigs [70]. These knock-in alleles are orthologous to the clinically relevant FAP mutations occurring at human APC codons 1061 and 1309 [48, 49]. A*PC* ^*1311/+*^ pigs presented with polyps in the colon and rectum at one year of age; aberrant crypt foci (ACF) were detectable in the colon. A similar pathology occurs during the development of FAP in (young) human patients, signifying that the pig is also a suitable model for the study of this type of colorectal cancer.

## 4. Compound* Apc* Models Recapitulate the Pathology of FAP More Precisely

Cancer is considered to be a multistage process, requiring the cumulative acquisition and integration of a number of cellular, genomic, and possibly epigenomic alterations operationally grouped into several defining hallmarks [71]. The eventual outcome is cellular transformation, clonal expansion, and tumor formation. Important as it is, mutation of* Apc* is not the sole criterion required for colorectal tumorigenesis.* Apc* mutant mice can be crossed with mice “knocked out,” or deficient, at other loci or with other alleles to generate compound animals. Combination of* Apc* mutant backgrounds with mouse strains knockout or defective in key DNA repair genes can be used to recapitulate the FAP phenotype more thoroughly and extend the versatility of this colorectal cancer model. Any changes of the tumor phenotype on the* Apc* mouse background provide a biological readout for assessing the effects of knockout, or overexpression, of other genes. Such crosses are instructive for assessing the relative contribution of known or newly discovered genes to the development of colorectal cancer.

The list of* Apc* compound knockouts is extensive and continually growing. We will briefly discuss some examples and highlight key insights that helped to further our understanding of the biological mechanisms involved in colorectal cancer. (1)* Apc* ^Δ*716/+*^ *;Smad4* ^*+/*−^ heterozygote mice develop intestinal polyps which progress more quickly to invasive adenocarcinoma [72]. Although* Apc* loss of function is required for adenoma formation, loss of function of other genes, such as* Smad4*, is necessary for malignant progression. (2) Certain genes exert regional-specific effects on polyposis along the intestinal tract.* Apc* ^Δ*716/+*^ *;Cdx* ^*+/*−^ mice develop 6-fold more polyps in their colons but 9-fold less polyps in their small intestines compared to* Apc* ^Δ*716/+*^ mice [73]. The increase in colonic polyps is caused by upregulation of* mTor* signaling which thus presents a possible therapeutic target. (3)* Apc* ^*Min/+*^ *;BubR1* ^*+/*−^ mice also develop more colonic polyps, by a factor of ten, than* Apc* ^*Min/+*^ mice. It was concluded that both* Apc* and* BubR1* functionally interact in regulating metaphase-anaphase transition [74]. Haploinsufficiency of these proteins in the compound heterozygotes increased chromosomal instability as a function of spindle checkpoint deregulation which accelerated cancer development and progression. (4) Specific deletion of both* Apc* and* Myc* was achieved in the small intestine using the* Apc* ^*580S*^ allele crossed to an* Ah-Cre* ^*+*^ *;Myc* ^*fl/fl*^ compound mouse [75]. Expression array analyses of tumor tissues from these mice revealed that, upon* Apc* loss,* Myc* becomes a critical mediator of concomitant neoplasia and highlighted the potential of* Myc* as a possible therapeutic target in intestinal tumorigenesis. (5) Haploinsufficiency for* Blm* on an* Apc* ^*Min/+*^ background increased tumor formation about 2-fold [76]. Genomic analyses indicated that increased tumor formation was due to an increase in somatic recombination, which facilitated inactivation of the wild-type* Apc* allele by interchromosomal recombination leading to LOH (see below). These observations are of relevance to human populations, with similar conclusions being reached about carriers of the* BLM* ^*Ash*^ mutation and their susceptibility to colorectal cancer [77].

## 5. Models of Lynch Syndrome/Hereditary Nonpolyposis Colon Cancer

Lynch syndrome is an autosomal dominant predisposition to colorectal cancer that was first described over a century ago [78] and comprehensively studied over many years, by Lynch and colleagues, among others [79, 80]. It is the most common cancer predisposition syndrome in the human population and has been estimated to occur at an incidence of 1 in 660 individuals, although, given that screening methods are not 100% inclusive, the actual incidence is probably lower [81]. Consensus criteria for Lynch syndrome diagnosis were internationally agreed upon [82] and have been refined subsequently to reflect the better understanding of this disease at both the clinical and mechanistic levels [83–85]. Patients develop early-onset colorectal cancers and a subset of these is also associated with extracolonic tumors at sites including the stomach, small intestine, ovaries, and endometrium [40, 83, 84]. Individuals predisposed to Lynch syndrome carry heterozygous mutations in various genes of the MMR pathway, most notably* MSH2*,* MSH6*,* MLH1*, and* PMS2* [40, 84, 86]. MMR constitutes a postreplicative DNA repair system and the mechanistic details of this pathway have been reviewed elsewhere [87]. Around 15% of sporadic colorectal cancers also display MMR defects; hereditary and sporadic tumors can be differentially stratified on the basis of various molecular and morphological criteria [86]. Cells lose their wild-type MMR allele by various somatic means, facilitating tumor development due to the increased mutation rates normally suppressed by the MMR system [40, 85, 86]. The resultant mutator phenotype, a genetic hallmark coupled to MSI, provides the environment for the accumulation of multiple secondary mutations. Changes in the length of normally stable short DNA repeat sequences (microsatellites) are now standard diagnostic indicators of the MSI classically associated with MMR defects [41, 42]. Tumor tissues from most Lynch syndrome cases associated with MMR defects display MSI [85, 86].

Knockout mouse lines have been generated for all of the genes known to comprise the MMR system:* Msh2* [46, 88],* Msh3* and* Msh6* [89, 90],* Mlh1* [91, 92],* Pms1* and* Pms2* [93],* Mlh3* [94], and* Exo1* [95]. Multiple lines have been made for some of these genes; for example, there are at least nine different alleles for the* Msh2* gene [96]. Although knockout lines have been generated for* Msh4* and* Msh5*, which are acknowledged members of the MMR family, these genes do have traditional functions in postreplicative DNA repair but instead are associated with defects in meiosis [97, 98]. The severity of tumor phenotypes exhibited by mice deficient in various genetic components of MMR correlates well overall with the known roles and contributions of these genes to Lynch syndrome. Mice deficient in* Msh2* and* Mlh1* develop more tumors more quickly and have the shortest median survival times than mice deficient in other aspects of MMR [24, 46, 88, 92].* MSH2* and* MLH1* are central to MMR function and are mutated with the highest frequencies in Lynch syndrome tumors, indicative of their pivotal importance in this DNA repair pathway, and the inherent selection required for their inactivation leading to tumorigenesis.

Although knockout mice for* Msh2*,* Mlh1*, and* Msh6* gene function develop gastrointestinal tumors, most actually die from lymphomas and thymomas [24, 46, 88–90, 92]. This was long argued to be a weakness of these models, but this perception has been revised with the recent identification of patients who are constitutively defective in MMR; that is, they possess biallelic inactivating mutations in* MLH1*,* MSH2*,* MSH6*, or* PMS2* [99]. These patients present with early-onset hematological and brain malignancies. In retrospect, the phenotypes of conventional homozygous knockout mice have actually proven to be good models for patients who are constitutively defective in MMR, as opposed to heterozygous carriers at risk of Lynch syndrome.

Conditional mouse models have also been created for the MMR genes most prominently involved in Lynch syndrome. A floxed allele of* Msh2* has been developed that, in combination with an intestinal-specific Cre recombinase transgene, facilitates restriction of* Msh2* inactivation to the intestinal epithelium [100]. In this model, designated* VCMsh2* ^*loxP*^ (*Villin-Cre;Msh2* ^*loxP/loxP*^) animals do not present with lymphomas. Tumorigenesis is restricted to the intestinal compartment and mice develop 1-2 adenomas or adenocarcinomas within the first year. The* VCMsh2* ^*loxP*^ line was combined with* Msh2*-null or* Msh2* ^*G674A*^ alleles to generate allelic phase mutants. These animals were used to investigate the therapeutic potential of specific drugs on colorectal tumorigenesis in an* in vivo* setting. A similar model (*Mlh1* ^*flox/flox*^) has also been generated for conditional inactivation of* Mlh1* [101]. Conditional targeting has been used to ablate Msh2 expression in the crypt base columnar stem cells (CBCs) of the mouse intestinal crypt [102]. The* Lgr5-CreERT2* mouse line, originally developed by Barker and colleagues [103], was sequentially crossed with* Msh*-null and* Msh* floxed lines to generate* Lgr5-CreERT2;Msh2* ^*flox/*−^ mice. By judicious administration of tamoxifen, it was possible to generate mosaic* Msh2*-inactivation within a small field of CBCs, thus more closely mimicking the mutational developments that occur during early stages of Lynch syndrome. On average, tamoxifen treated* Lgr5-CreERT2;Msh2* ^*flox/*−^ mice developed invasive adenocarcinomas after 19 months [102]. All tumors were negative for Msh2. It is clear that when directed with precision and studied in context, mouse models are useful systems for studying tumor suppression by the MMR system and investigating its role in colorectal tumorigenesis.

## 6. Models of Sporadic Tumorigenesis

Most colorectal cancers developed are sporadic in nature and develop without selective pressure from genetic predisposition, lacking germline heterozygosity in any inherited mutant allele. The fundamental challenge of developing sporadic models of colorectal cancer, or any other cancer for that matter, is in the adaption of available genetic systems to control biological processes in a stochastic nonheritable way. The final goal is to orchestrate the formation of defined tumor phenotypes in specific tissues under essentially random, yet controllable, conditions. Many sporadic tumor models involve the Cre-*loxP* or FLP-FRT systems. They are routinely designed around a floxed tumor suppressor gene or a floxed latent allele of an activated oncogene and require inducible or stochastically regulated expression of Cre recombinase to direct deletion (tumor suppressor) or activation (oncogenes) of the target gene [3, 104]. Although many sporadic models of tumorigenesis are variations on this theme, two separate models of sporadic Lynch syndrome have been recently reported that uniquely feature an out-of-frame Cre containing a mononucleotide microsatellite sequence [105, 106]. Expression of wild-type Cre is dependent on the acquisition of a frameshift reversion within the mononucleotide sequence, thus restoring an open reading frame. This may, or may not, be due to acquisition of an MSI phenotype. The sporadic expression of wild-type Cre subsequently drives deletion or activation of other floxed alleles.

The RAS family of genes is somatically mutated in about 30% of all tumors and around 50% of colorectal cancers develop mutations specifically in* KRAS*, the majority occurring at codon 12 [39]. One of the earlier models of sporadic cancer featured a latent allele of the* K-ras G12D* activating mutation (*K-ras* ^*LA*^) [107]. Activation of this allele was solely dependent on intrachromosomal recombination between contiguous regions of the genetically restructured endogenous* K-ras* locus; no other exogenous agent, such as Cre, was required. Although tumors developed in the lungs of these mice and all mutant animals developed ACF in the colon, intestinal tumors were not observed. This correlates with the detection of* KRAS* mutations in ACF in humans [108]. A similar strategy has been used to direct sporadic activation of a latent allele of *β-catenin* which features a clinically relevant Ser→Phe mutation at codon 37. This mutation ablates a Gsk-3-*β* phosphorylation site, important for *β*-catenin regulation, and, after intrachromosomal recombination, results in expression of an oncogenic form of *β*-catenin [109]. In this model, sporadically activated *β*-catenin was sufficient for tumor initiation but did not lead to malignant progression. Sporadic multifocal lesions developed only in the stomach; adenomas were not detected in any tissue. This is in contrast to other mouse models of activated *β*-catenin signaling which demonstrate a clear association between expression of oncogenic forms of *β*-catenin and intestinal tumorigenesis [110, 111].

The* K-Ras* ^*LA*^ model described above [107] was subsequently modified by the incorporation of a Lox-Stop-Lox (LSL) cassette. This enabled activation of the latent* Ras* ^*G12D*^ allele by Cre recombinase, administrable by various platforms, including adenoviral infection, which removed the intervening LSL cassette and restored transcriptional integrity of the mutant allele at the endogenous locus [112]. Although the LSL* K-Ras* ^*G12D*^ model was initially used to investigate* Ras* involvement in lung tumorigenesis, it was quickly coopted for studies in other cancers including leukemia [113], squamous cell carcinoma [114], and of course colorectal cancer [115, 116]. The models demonstrated that oncogenic* K-ras* induced ACF in the colon, but progression to microadenoma was determined by regional-specific factors within this tissue; ACF in the proximal colon progressed to adenoma whereas those in the distal colon did not [115]. Activated* K-ras* affected proliferation and differentiation in the colonic epithelium of nonneoplastic tissues by signaling through Mek but in itself was not sufficient to drive neoplasia [116].

The LSL* K-Ras* ^*G12D*^ allele has been incorporated into yet another mouse model of sporadic colon cancer, one that successfully recapitulates the adenoma-carcinoma-metastasis trajectory common to human colon cancers [117]. This is an important achievement, as it is still challenging to faithfully model metastatic spread of intestinal cancer. The genetic units of this model entail the LSL* K-Ras* ^*G12D*^ and* Apc* ^Δ*580*^ alleles and a Cre-expressing adenovirus (Adeno-Cre). Surgical procedures were used to restrict Adeno-Cre delivery to the mouse colon, resulting in an average tumor burden of 3.6 lesions per animal, which contrasts with the tumor multiplicities of the original models: 0 for LSL* K-Ras* ^*G12D*^ [112] and ~120 for* Apc* ^Δ*580*^ [32]. The limited tumor burden increased animal survival, a factor undoubtedly contributing to the successful metastatic spread of tumors from the colon to the liver, which started around 24 weeks after infection with Adeno-Cre. Insights from this model are that activated* K-ras* can accelerate tumor progression in conjunction with an established* Apc* mutation and that* K-Ras* ^*G12D*^ has also the capacity to promote metastatic spread, when expressed against the appropriate cellular background.

## 7. RecQ Helicases: Guardians of the Genome

RecQ helicases are evolutionarily conserved from bacteria to humans and have multiple, sometimes overlapping, roles in DNA metabolism including replication, recombination, and repair [118]. The five known homologs of the mammalian RecQ family, RECQL1, BLM, WRN, RECQL4, and RECQL5, play pivotal roles in the maintenance of genomic stability and cellular homeostasis. Mammalian RecQs can not only form heterologous complexes with other family members but also interact with many other proteins involved in various DNA maintenance/repair pathways [119].* BLM*,* WRN*, and* RECQL4* are linked to monogenic genetic diseases characterized by genome instability, premature aging, and cancer predisposition [118, 120].

Bloom's syndrome (BS) is a hereditary disease characterized by a predisposition to various types of cancers that first present at a mean age of 24 years [121]. Characteristic phenotypes manifested by BS patients are severe growth retardation and a high susceptibility for cancers of all types [121, 122]. The* BLM* gene is mutated in individuals with BS [123]. The Groden laboratory has studied BLM (and WRN) for many years and we use BS as a paradigm for understanding how DNA repair deficiency impacts both growth and cancer. BLM responds to DNA damage-induced stress sustained during DNA metabolism including the restart/repair of stalled and collapsed replication forks during DNA replication, the repair of interstrand cross-links, the resolution of Holliday junctions, and the suppression of aberrant homologous recombination [119, 120]. BLM also functions in telomere maintenance and is specifically involved in telomerase-independent telomere elongation in the alternative lengthening of telomeres (ALT) pathway [124–126]. Furthermore, our recently published studies have established a role for BLM in regulating rDNA metabolism [127, 128].

BLM deficiency results in major genomic instability—a hallmark of most cancers and a factor that escalates the cancer frequency in those with BS. BLM interacts with many other DNA damage response proteins, including BRCA1, MLH1, MSH2, MSH6, p53, RAD51, topoisomerase II*α*, and WRN [129–134]. Some of these partners function as sensors and transducers in DNA damage response pathways, colocalize with BLM in the nucleolus, and physically associate with BLM to facilitate repair functions [126–128, 134–137]. Consistent with its role in DNA repair, BLM deficiency results in the formation of aberrant chromosomal structures and increased sister chromatid exchanges (SCE) [120, 138].

## 8. RecQ Mutant Mouse Models and Colorectal Cancer

The role of disrupted homologous recombination (HR) in human cancer susceptibility is well established by studies of tumor incidence in BS, where loss of the BLM helicase increases inter- and intrachromosomal recombination [120] and the high incidence of breast and ovarian cancer in carriers of* BRCA1* mutation, where loss of BRCA1 suppresses HR and impedes DNA double-strand break repair [139]. Similarly, decreased DNA repair capacity and/or dysregulated HR in mouse models of cancer lead to increased tumor susceptibility, although some experiments suggest that such alterations can inhibit tumor formation [140]. Published studies show that intestinal tumor number and histological characteristics in mouse models vary when DNA repair proficiency or chromosome stability varies [74, 76, 140, 141].

To date, six different mouse models of Blm have been reported in the literature. In four of these models,* Blm* ^*tm1Grd*^ (*Blm* ^*Cin*^),* Blm* ^*tm1Ches*^,* Blm* ^*tm2Brd*^ (*Blm* ^*m2*^), and* Blm* ^*tm3Ches*^, homozygosity for the mutated* Blm* allele results in embryonic lethality [76, 142–144]. The *Blm* ^*tm*3*Brd*^ (*Blm* ^*m3*^) allele, also generated by Luo and colleagues, was originally reported as a null mutation which ablated Blm expression [143]. However, it has since been recharacterized as a hypomorphic allele which expresses Blm at approximately 25% of wild-type levels [144]. The* Blm* ^*tm4Ches*^ model consists of a conditionally floxed allele which facilitates tissue-specific ablation of* Blm* function when crossed onto the appropriate Cre recombinase background, thus circumventing the developmental issues of embryonic lethality [145, 146]. Cell lines and tissues from the above Blm models exhibit increased levels of DNA damage and SCE [76, 142–146] underscoring the roles of* BLM/Blm* in the maintenance of genomic stability. Previous studies using the* Apc* ^*Min*^ mouse model of intestinal tumorigenesis demonstrate that increased tumor dysplasia and tumor number occur in (heterozygous)* Blm* ^*Cin/+*^ *;Apc* ^*Min/+*^ (Figure 1(c)) or (hypomorphic)* Blm* ^*m3/m3*^ *;Apc* ^*Min/+*^ mice [76, 143]. These changes in tumor biology are driven by increased rates of homologous recombination which facilitates LOH of the remaining wild-type* Apc* allele. In contrast,* Blm* haploinsufficiency had no impact on tumor development, progression, or regression in a* Ccsp/Fgf-10* transgenic model which overexpresses the growth factor Fgf-10 under control of the lung-specific Clara cell secretory protein (*Ccsp*) promoter (Figure 1(d)) (Boivin & Groden, personal communication). There were no significant differences in the numbers, size, and histologic grade of lung adenomas between transgenic* Fgf-10* and* Blm* ^*Cin/+*^ *;Fgf-10* mice. Indeed, the* Apc* ^*Min/+*^ intestinal and* Fgf-10* lung models of adenoma formation may differ in their underlying mechanistic basis, but there are undoubtedly tissue-specific contributions affecting the emergent tumor phenotype(s) when Blm levels are genetically modulated in specific cellular compartments.

Werner syndrome (WS) is a segmental progeroid disease that causes premature aging in affected individuals [147, 148]. Patients have an elevated risk for age-related diseases including atherosclerotic cardiovascular disease and a wide range of cancers [149]. Defects in the Werner gene (*WRN*) are the underlying cause of WS. Similar to BLM, WRN is involved in mitotic recombination and is also important in the ALT pathway of telomere maintenance [135, 150]. A small proportion of WS patients develop gastrointestinal tumors, and* WRN* is epigenetically silenced by promoter methylation in colorectal cancer [151, 152]. Specific* WRN* polymorphisms have been investigated in GWAS studies for susceptibility to colorectal cancer, but of these only the WRN Cys1367Arg variant was associated with increased risk [153, 154]. Mouse models have been generated for WRN, but* Wrn*-deficient mice do not recapitulate the phenotype of WS patients [155, 156].* Wrn* ^−*/*−^ mice demonstrate neither premature aging nor develop tumors. Overall they appear phenotypically normal, but this may, in part, be due to the fact that mouse telomeres are considerably longer than human. Indeed, when the* Wrn* ^−*/*−^ knockout background is crossed with the* Terc-* (telomerase RNA template) deficient mouse model and/or the hypomorphic* Blm* ^*m3*^ model, it accelerates the onset of several phenotypic aspects characteristic of later generation* Terc* ^−*/*−^ animals by 2-3 generations [157], suggesting a role for* Wrn* and* Blm* in aging. To our knowledge, the* Wrn* ^−*/*−^ knockout has yet to be crossed with the* Apc* ^*Min*^ model, so it remains unknown what influence* Wrn*-deficiency will have on intestinal tumorigenesis in this setting. Recql5 is the only other RecQ family member that has been crossed with the* Apc* ^*Min*^ model.* Recql5* ^−*/*−^ *;Apc* ^*Min/*+^ mice develop twofold more intestinal adenomas than control* Apc* ^*Min/+*^ cohorts [158]. Given the known, overlapping roles of the mammalian RecQ family in maintaining genomic stability, it will be no surprise if other members of this group modify the intestinal tumor phenotype of the* Apc* ^*Min*^ model.

## 9. *In Vivo* Manipulation of BLM Levels Modulates Intestinal Tumorigenesis

Several lines of evidence indicate that* Blm* dosage is critical for controlling the onset of tumorigenesis in mice. Mouse models demonstrate that chromosomal instability directly correlates with the levels of Blm; as Blm decreases, genomic instability and tumor burden increase [76, 143, 144].* Blm* ^*Cin/+*^ mice develop lymphoma earlier than wild-type litter-mates when challenged with murine leukemia virus [76]. Furthermore, haploinsufficiency for* Blm* on the C57Bl/6J* Apc* ^*Min/+*^ background increases spontaneous adenoma formation and dysplasia facilitated by an increase in HR which leads to LOH and hence loss of the wild-type* Apc* allele. These observations correlate with studies on human carriers of specific* BLM* mutations and their subsequent susceptibilities to colorectal cancer [77]. Homozygous* Blm* ^*m3/m3*^ mice develop a wide spectrum of different tumors by age of 20 months, analogous to those presented by BS patients [143]. Additionally, the hypomorphic* Blm* ^*m3/m3*^ mutant accelerates onset of several phenotypic aspects characteristic of later generation* Terc*-deficient mice by 2-3 generations, including reduced life span, increased apoptosis of epithelial crypt cells, and increased chromosome end-to-end fusions [157].

**Figure 2:**
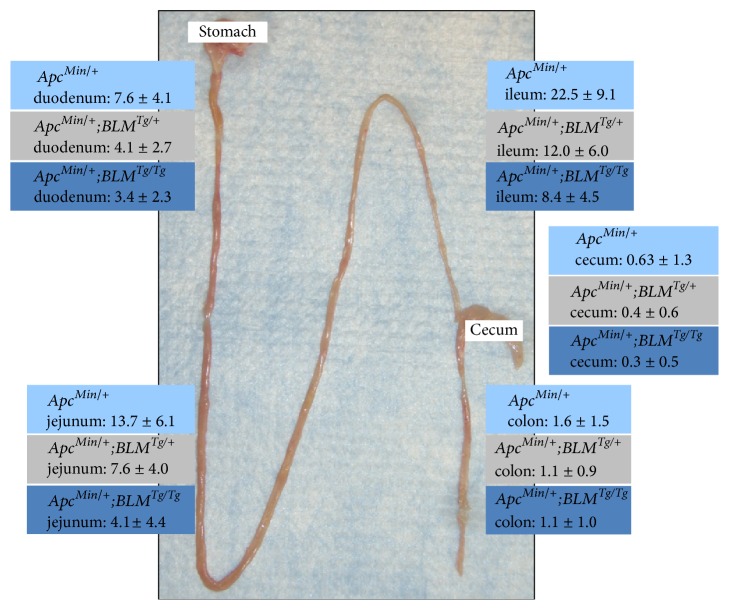
*BLM* ^*Tg*^ reduces intestinal polyp numbers by ~2-fold in* Apc* ^*Min/+*^ mice. Polyps counts (mean ± *σ*) for different regions of the gastrointestinal tract are shown for* Apc* ^*Min/+*^,* Apc* ^*Min/+*^ *;BLM* ^*Tg/+*^, and* Apc* ^*Min/+*^ *;BLM* ^*Tg/Tg*^ mice.* BLM* ^*Tg*^ dose-dependent suppression of adenoma numbers is most evident in the jejunum and ileum.* BLM* ^*Tg*^ does not change tumor spectrum or location.

Transgenic mouse models have been developed that overexpress DNA repair genes. Some models develop tumor-resistant phenotypes with increases in animal survival and/or cancer-free survival and significant increases in animal longevity [23, 159–162]. These reports confirm that overexpression of genes involved in DNA repair not only has anti-tumorigenic effects but also positively impacts the myriad of pathways that contribute to organismal aging. We hypothesized that if halving* Blm* gene dosage increased predisposition to tumorigenesis, overexpression would conversely decrease tumor susceptibility and consequently develop a transgenic mouse model that expresses human* BLM* under control of the* PGK* promoter (*BLM* ^*Tg*^). This transgene rescues the embryonic lethality of* Blm* ^*Cin/Cin*^ knockout mice, indicating that BLM expression is appropriately regulated, within the physiological context of our model, to direct normal development in* Blm*-null mice [141]. Given the demonstrated relationship between low or absent expression levels of BLM/Blm and cancer, we investigated whether constitutive overexpression of BLM attenuated adenoma formation in our* Apc* ^*Min*^ mouse model of intestinal tumorigenesis. Although there is a 50% reduction in the number of intestinal adenomas that spontaneously arise in* Apc* ^*Min/+*^ *;BLM* ^*Tg*^ mice, there is no difference in tumor pathology (Figure 1(e)). Suppression of adenoma formation by* BLM* ^*Tg*^ was most evident in the jejunal and ileac segments of the gastrointestinal tract (Figure 2) which is not surprising, as these regions comprise the predominant site of adenoma formation in* Apc* ^*Min/+*^ [163]. Adenomas were not observed in* BLM* ^*Tg*^ or wild-type mice.

Given the role of* BLM* in maintaining genomic integrity [118–120], we hypothesized that* BLM* ^*Tg*^ modulated tumorigenesis in* Apc* ^*Min/+*^ mice by suppressing HR, thus reducing the rate of LOH and hence loss of the wild-type* Apc* allele and possibly the rate of secondary genomic events that additionally affect genome stability. To investigate further the mechanism of intestinal tumor reduction by BLM overexpression in* Apc* ^*Min/+*^ *;BLM* ^*Tg*^ mice, we used the pink-eyed unstable (*p* ^*un*^) model as an* in vivo* reporter for measuring levels of HR. In this serendipitous model, which originates from a naturally occurring mutation, a somatic intrachromosomal deletion within the mouse *p* gene restores melanin production in the otherwise transparent cells of the retinal pigment epithelium (RPE), generating a clone of brown cells or eyespot [164]. Deletion events occur spontaneously and are absolutely dependent on HR. Thus, the number of RPE eyespots is an* in vivo* surrogate for levels of HR within the tissue (Figures 1(f) and 1(g)). The* p* ^*un*^ model has previously been used to demonstrate the opposing roles that* Blm* and* Brca1* play in HR [165]. A twofold reduction of eyespots in RPE cells of* p* ^*un/un*^ *;BLM* ^*Tg*^ mice suggests that* BLM* ^*Tg*^ directly modulates HR in this tissue [141]. Our interpretation of the observed reduction in adenoma numbers in the* Apc* ^*Min/+*^ *;BLM* ^*Tg*^ model is that elevated levels of BLM/Blm reduce HR in the intestinal epithelia, thus suppressing LOH and hence loss of the wild-type* Apc* allele.

It has been observed that when the* Apc* ^*Min*^ allele is combined with mismatch repair- (MMR-) null mouse models, either* Mlh1* ^−*/*−^ or* Msh2* ^−*/*−^, the mechanism of* Apc* inactivation changes from that of LOH to intragenic mutation. Analyses of adenomas from* Mlh1* ^−*/*−^ *;Apc* ^*Min/+*^ and* Msh2* ^−*/*−^ *;Apc* ^*Min/+*^ mice demonstrated intragenic (point) mutation of the wild-type* Apc* allele in 81% and 85% of cases, respectively [46, 52, 53]. This shift in the mechanism of* Apc* inactivation is due to the characteristic mutator phenotypes inherent to these models of MMR deficiency. Given the known roles of* BLM/Blm* in HR, we investigated if our* BLM* ^*Tg*^ could likewise reduce intestinal adenoma burdens in an* Apc* ^*Min/+*^ model that was not dependent on LOH as a second-hit mechanism of inactivation. When the* Apc* ^*Min/+*^ *;BLM* ^*Tg*^ model was crossed onto a* Msh2* ^Δ*7N*^ (MMR-null) background there were no significant differences in intestinal adenoma numbers (Figure 1(h)) between* Apc* ^*Min/+*^ *;BLM* ^*Tg*^ *;Msh2* ^Δ*7N/*Δ*7N*^ and* Apc* ^*Min/+*^ *;Msh2* ^Δ*7N/*Δ*7N*^ mice [141]. Thus, inactivation of the wild-type* Apc* allele by point mutation, due to innate MMR deficiency, rather than by LOH, ablates the protective, suppressive effect of the* BLM* ^*Tg*^ on intestinal adenoma formation. Although indirect, this observation supports our hypothesis that genetic upregulation of BLM/Blm expression reduces HR in the intestinal epithelia, thus suppressing LOH and hence loss of the wild-type* Apc* allele. Collectively, the data suggest that HR-dependent DNA double-strand break repair capacity can be modulated* in vivo* to alter tumor susceptibility and that perhaps levels of specific DNA repair proteins may be titrated to achieve positive therapeutic outcomes in the context of specific hereditary cancer syndromes, exemplified by FAP.

Cancer (and aging) represents complex phenotypes that develop through the integrated output of numerous biological pathways. It is possible that variation in BLM levels within normal human populations could also confer differential protection from/susceptibility to tumor formation in different individuals. Specific alleles of* BLM* have been associated with human cancers: BLM P868L (rs11852361) with colorectal cancer (odds ratio = 1.29, 95% CI, 1.02–1.64: *p* = 0.04) and BLM rs2532105 with breast cancer (odds ratio = 2.0, 95% CI: 1.2–3.3, *p* ≤ 0.05), respectively [153, 166]. Although the observation of decreased adenoma numbers in our* Apc* ^*Min/+*^ *;BLM* ^*Tg*^ model is associated with overall increased BLM expression, the example of BLM P868L in colorectal cancer [153] suggests that functional variation within* BLM* alleles might be equally important in contributing to a tumor-resistant/susceptible phenotype. Indeed,* in vitro* analyses of hypomorphic BLM variants, including BLM P868L, have demonstrated that polymorphisms/mutations within the human population have biological consequences for BLM function [167]. It is possible that certain long-lived humans may inherit superior functional variants of* BLM* alleles with elevated expression that contribute to increased genomic stability, protecting against tumorigenesis and thus extending life span. Although loss-of-function* BLM* alleles have been associated with human cancers [153, 166] genome-wide association studies have yet to identify alleles associated with longevity [168]. The early onset of tumorigenesis in BS individuals makes it difficult to resolve the functional protective role(s) that BLM may have in organismal aging [120, 121]. This may in part be due to the highly selective nature of markers chosen for genome-wide association studies and suggests that understanding the effects of specific* BLM* alleles and/or associated haplotypes on tumor repression in humans will remain a future challenge. Understanding the mechanism by which BLM attenuates tumor susceptibility will aid in our fundamental understanding of its roles in maintaining genomic stability and suggest new strategies for cancer prevention involving direct regulation of DNA repair pathways.

## 10. Therapeutic Insights from Mouse Models

Mouse models have proven good preclinical platforms for assessing the potential efficacy of chemopreventive and chemotherapeutic drugs against colorectal cancer. An unmistakable advantage is the capability to knock in the equivalent of clinically relevant human mutations and study their subsequent effects on tumorigenesis. A trio of common missense mutations identified in the* MSH2*,* MLH1*, and* MSH6* genes of Lynch syndrome patients have been knocked into mouse backgrounds, generating models corresponding to the* Msh2* ^*G674A*^ [169],* Mlh1* ^*G67R*^ [170], and* Msh6* ^*T1217D*^ [171] mutations. These missense models establish additional physiological contexts for recapitulating and unraveling the tumorigenic processes leading to Lynch syndrome and represent genetic systems that facilitate the* in vivo* analyses of clinically important mutations. The* Msh2* ^*G674A*^,* Mlh1* ^*G67R*^, and* Msh6* ^*T1217D*^ mutations have been characterized as separation-of-function alleles and in these animals the normally intertwined processes of mismatch DNA repair and the apoptotic response to DNA damage have been genetically severed, resulting in distinctive phenotypes. Although mutant mice still demonstrated strong tumor dispositions, their normal apoptotic responses to DNA damaging agents such as* N*-methyl-*N*′-nitro-*N*-nitrosoguanidine (MNNG) and cisplatin were intact [169–171]. MMR-deficient cells are traditionally resistant to these types of compounds. However, a recent report conflicts with the finding that* Msh2* ^*G674A*^ is a separation-of-function allele. Oligonucleotide-directed mutagenesis was used to screen* MSH2* variants of uncertain significance (VUS) in mouse embryonic stem cells hemizygous for* Msh2* (*Msh2* ^*+PUR/*Δ^) [172]. In this system,* Msh2* ^*G674A*^ conferred partial resistance to alkylating agents. It is possible that the* Msh2* ^*G674A*^ variant functions differently in human and mouse and that differences between the* in vitro* and* in vivo* experimental settings could lead to conflicting biological outcomes.

When the* VCMsh2* ^*loxP*^ line was combined with* Msh2-*null and* Msh2* ^*G674A*^ models to generate allelic phase mutants, only tumors from* VCMsh2* ^*loxP/G674A*^ animals were responsive to treatment with FOLFOX (folinic acid; fluorouracil; oxaliplatin), a chemotherapeutic regime used to treat late-stage colorectal cancer [100]. An obvious implication from these models is that Lynch syndrome patients with certain missense mutations will prove susceptible to treatment with conventional chemotherapeutic agents and this suggests additional criteria that may prove useful for stratifying Lynch syndrome patients with respect to optimal treatment. Treatment of* Lgr5-CreERT2;Msh2* ^*flox/*−^ mice with temozolomide (TMZ), a methylating chemotherapeutic agent, provides corroborating data for stratifying therapeutic regimes. TMZ promoted expansion of* Msh2*-deficient crypts over 5-fold in* Lgr5-CreERT2;Msh2* ^*flox/*−^ mice, consistent with the interpretation that* Msh2*-deficient CBCs develop a competitive growth advantage in the crypt stem cell niche [102]. This is congruent with the proposed biased drift model of stem cell dynamics that governs the mutational trajectories of CBCs after acquisition, or induction, of oncogenic mutations [173]. Moreover, drug treatment accelerated intestinal tumor development in* Lgr5-CreERT2;Msh2* ^*flox/*−^ mice, most likely caused by the increased mutational load from TMZ and compounded by the MSI phenotype of* Msh2*-deficient CBCs [102]. In conclusion, Lynch syndrome patients should not be exposed to TMZ, which is paradoxically a strong risk factor for tumor development, as it selects for and causes expansion of highly tumorigenic* Msh2*-deficient cells in this therapeutic setting.

Various FAP and Lynch syndrome mutant mouse lines have been employed over many years to study the potential of nonsteroidal anti-inflammatory drugs (NSAIDs), a structurally diverse family of compounds, as chemopreventive options in cancer treatment [174, 175]. Epidemiological studies have clearly reported an inverse relationship between the use of certain NSAIDs and the incidence of colorectal cancers [176, 177] and mouse models remain appropriate experimental systems for investigating the anti-tumorigenic mechanisms of these compounds. Although it is accepted that NSAIDs interrupt arachidonic acid metabolism* via* inhibition of COX enzymes, thus modulating the synthesis of prostaglandins [178], they also exhibit pleiotropic effects on other cellular pathways.

Aspirin suppresses the MSI in MMR-deficient human colon tumor cell lines via a genetic selection that appears to enhance apoptosis in critically unstable cells [179]. The long-term outcome is a cell population that has a persistent deficiency in MMR but has paradoxically acquired a largely microsatellite stable (MSS) phenotype. Remarkably, the selection for MSS in cells that were MMR-deficient was independent of the* COX1* or* COX2* genes [179]. Nitric oxide-donating aspirin (NO-aspirin) also suppressed MSI in MMR-deficient cell lines but at concentrations 300- to 3000-fold less than aspirin [180]. We hypothesized that treatment with aspirin and NO-aspirin would delay and/or prevent tumorigenesis in Lynch syndrome. When aspirin and NO-aspirin were used to treat a mouse model of Lynch syndrome (*Msh2* ^*flox/flox*^ *;Villin-Cre*) it was observed that both reagents delayed onset of tumorigenesis and increased animal survival [181]. Furthermore, aspirin appeared to partially stabilize tumor MSI in this model, possibly through an apoptotic process that eliminated critically unstable cells, thus attenuating, but not completely reversing, the intrinsic mutator phenotype. If we can identify and understand important signaling pathways that are important in determining the chemopreventive properties of various NSAIDs, they may reveal new opportunities for alternative, more focused therapies for the treatment of colorectal cancer.

The Colorectal Adenoma/Carcinoma Prevention Program (CAPP) has examined the potential of aspirin to reduce colorectal neoplasia in Lynch syndrome carriers. The initial CAPP report concluded that 4-year exposure to aspirin did not significantly reduce the incidence of neoplasia [182], although mouse studies clearly suggested that long-term exposure to aspirin was required for chemopreventive benefits. Moreover, a recent clinical analysis reported that regular aspirin use was associated with a lower risk of cancer-specific mortality in individuals already diagnosed with colorectal cancer [183]. Similar observations have been reported for the chemopreventive role of aspirin in breast cancer [184, 185]. Together, these results suggested that the chemopreventive benefits of aspirin might only manifest after long-term continuous administration. The updated report from the CAPP trial indicates that, perhaps not unexpectedly, the benefits of aspirin for Lynch syndrome patients only begin to appear after 5 years of exposure [186]. The end-point for these studies was Lynch syndrome cancers detected during yearly colonoscopy screens. Analyses suggest that the proportion of patients with Lynch syndrome tumors dramatically decreases in the aspirin-treated cohort. On consideration of the combined cellular, mouse, and clinical studies it appears that aspirin presents a particularly promising chemopreventive agent for colorectal cancer. Indeed, The United States Preventive Services Task Force (USPSTF) originally recommended against the use of aspirin for the prevention of colorectal cancer [187]. However, this assessment has been recently updated to include low-dose aspirin as a chemopreventive option for colorectal cancer (and cardiovascular disease) among adults aged between 50 and 69 [188].

## 11. Conclusion

Insights from* in vivo* modeling studies have had and will continue to have great impact on understanding the genetics of human colorectal tumors and the mechanisms that initiate and lead to their progression. They translate directly to understanding risk for colorectal cancer, showing us how we can exploit tumor mechanisms and personalize therapeutic interventions. Current modalities for colorectal cancer include therapies that target the VEGF (bevacizumab) and EGFR (cetuximab) pathways. Other druggable pathways are those for BRAF (vemurafenib) and of course Wnt/*β*-catenin signaling (OMP-18R5). Immunobased therapies, including those targeting CTLA4 and PD1, are also the focus of clinical trials for colorectal cancer. Adequate consideration of these regimes lies beyond the scope of this paper; they have been comprehensively reviewed elsewhere [189]. Chemopreventive trials in Lynch syndrome use high dose aspirin, while polyposis patients are treated prophylactically with celecoxib. Most therapies for colorectal tumors rely on leucovorin, 5-fluorouracil, and topoisomerase inhibitors (FOLFIRI) to treat locally and distantly invasive disease. However, other approaches are needed for improving the standard of care and for stratifying these approaches. Mouse models have been instrumental in demonstrating the fact that it is possible to modulate HR-dependent DNA double-strand break repair capacity* in vivo* to alter tumor susceptibility. Furthermore, it is conceivable that levels of specific DNA repair proteins may be titrated to achieve positive therapeutic outcomes in the context of specific hereditary cancer syndromes, exemplified by FAP. Whether this becomes eventually achievable in clinical settings remains a matter for speculation. However, development of these types of systems to target tumors more effectively may make it possible to augment the success of our current treatments for colorectal cancer.

## Figures and Tables

**Table 1:** Genetically engineered alleles of the mouse *Apc* gene.

*Apc* allele	Mutation	Polyp number	Pathology/comments	Ref
*Apc* ^*Min*/+^	Frameshift at codon 850 (ENU induced)	~30–>100	Polypoid, papillary and sessile adenomas; cystic crypts, no colonic ACF	[26]

*Apc* ^Δ*e*1–15^	Floxed exons 1–15, no protein	~210 ♀ ~150 ♂	Polypoid, papillary and sessile adenomas; cystic crypts, no colonic ACF	[27]

*Apc* ^15*lox*/+^	Floxed exon 15, frameshift at codon 650, truncation at 667	~185	Polypoid and sessile adenomas; cystic crypts; few colonic lesions	[28]

*Apc* ^*1322T/+*^	Neomycin cassette inserted into exon 15; stop at codon 1322	~200	Polyps predominantly in the first and second segments of the small intestine; few gastric and colonic polyps; polyps have reduced Wnt signaling relative to *Apc* ^*Min*^ polyps	[29]

*Apc* ^580*S*/+^	Floxed exon 14, frameshift at codon 580, truncation at 605	~7	Exposure to adenoviral-Cre; adenomas localized near anus	[30]

*Apc* ^Δ14/+^	Floxed exon 14, frameshift at codon 580, truncation at 605	~65	Polypoid and sessile adenomas; increase in colonic polyps, ACF, and rectal prolapses	[31]

*Apc* ^Δ580/+^	Floxed exon 14, frameshift at codon 580, truncation at 605	~120	Crossed to K14-Cre mouse line; polyploid and sessile adenomas; increase in colonic polyps, with additional abnormalities in the skin, thymus, and tooth	[32]

*Apc* ^*1638N*^	Neomycin cassette inserted into exon 15 in antisense; frameshift at codon 1638	~10	Colonic polyploid hyperplastic lesions, villous/tubulovillous adenomas; moderately to highly differentiated adenocarcinoma; rare gastric lesions	[33]

*Apc* ^1638*T*^	PGK-hygromycin cassette inserted in sense orientation; stop at codon 1638	0	Developmental abnormalities; growth retardation; absence of preputial glands	[34]

*Apc* ^Δ716/+^	Neomycin cassette inserted into exon 15; frameshift at codon 716	~254	Polypoid, papillary, and sessile adenomas; no colonic ACF	[35]

*Apc* ^1309/+^	Frameshift at codon 1309	~34	Polyps mainly in small intestine but also in the stomach and colon	[36]

*Apc* ^Δ474/+^	Neomycin cassette inserted into exon 9; duplication of exons 7–10; frameshift at codon 474	~122	Sessile-type polyps; rare mammary adenocarcinomas	[37]

*Apc* ^*neoF*^ *Apc* ^*neoR*^	Neomycin cassette inserted into intron 13 in both sense and antisense orientations	neoF ~ 1.00 neoR ~ 0.25	Dysplastic adenomas similar to those from *Apc* ^Δ716^ mice	[38]

A more extensive list of *Apc* mouse alleles can be found at http://www.informatics.jax.org/marker/phenotypes/MGI:88039.
